# Supplementary material for: Dietary Habits and Race Day Strategies among Flexitarian, Vegetarian, and Vegan Recreational Endurance Runners: A Cross-Sectional Investigation from The NURMI Study (Step 2)
Source: Nutrients. 2024 May 27;16(11):1647. doi: 10.3390/nu16111647 (PMC11174902; doi:10.3390/nu16111647)
Supplement: Supplementary file 1 [file nutrients-16-01647-s001.zip › nutrients-3011695-supplementary.pdf]

Table S1. Food frequency consumption of flexitarian, vegetarian, and vegan recreational distance runners.

	Total<br>100 % (146)	Flexitarian<br>23 % (34)	Vegetarian<br>34 % (50)	Vegan<br>43 % (62)	Statistics
**Total Grain Intake**	28.8 ± 12.8	29 ± 9.53	31.5 ± 14.6	26.6 ± 12.7	$F_{(2, 143)} = 1.42$; $p = 0.246$
**Whole Grains**	37.3 ± 17.1	37.3 ± 15.3	38.7 ± 18.3	36.1 ± 17.2	$F_{(2, 143)} = 0.16$; $p = 0.856$
**Cornflakes**	13 ± 10	13.4 ± 8.71	16.2 ± 11.4	10.3 ± 8.81	$F_{(2, 143)} = 4.80$; $p = 0.010$
**Legumes, Nuts, Pulses**	30.2 ± 13.8	28.3 ± 15.3	27.6 ± 14.1	33.4 ± 12.2	$F_{(2, 143)} = 3.66$; $p = 0.028$
**Fruit & Vegetables**	36.4 ± 12.2	34.9 ± 14.4	32.3 ± 10.6	40.6 ± 10.8	$F_{(2, 143)} = 8.54$; $p < 0.001$
**Dairy**	6.7 ± 11	14.3 ± 13.3	9.85 ± 11.5	/	$F_{(2, 143)} = 45.90$; $p < 0.001$
**Dairy Alternatives**	20.6 ± 15	20.3 ± 16.9	18.8 ± 14.1	22.2 ± 14.6	$F_{(2, 143)} = 0.96$; $p = 0.386$
**Meat & Fish**	1.26 ± 4.05	5.41 ± 7.01	/	/	$F_{(2, 143)} = 54.40$; $p < 0.001$
**Meat Alternatives**	8.45 ± 6.35	7 ± 6.35	8.25 ± 7.16	9.41 ± 5.54	$F_{(2, 143)} = 2.81$; $p = 0.064$
**Eggs**	3.7 ± 6.21	6.94 ± 6.25	6.08 ± 7.57	/	$F_{(2, 143)} = 33.73$; $p < 0.001$
**Oils & Spreads**	6.35 ± 7.14	7.69 ± 8.26	6.95 ± 7.4	5.13 ± 6.14	$F_{(2, 143)} = 1.15$; $p = 0.321$
**Snacks & Sweets**	10.5 ± 7.35	10.9 ± 7.41	11.9 ± 7.85	9.08 ± 6.75	$F_{(2, 143)} = 1.74$; $p = 0.180$
**Water & Unsweetened Tea**	41 ± 19.8	36.7 ± 15.9	41.6 ± 21.4	43 ± 20.2	$F_{(2, 143)} = 0.76$; $p = 0.470$
**Beverages**	13.8 ± 4.98	13.2 ± 4.4	14 ± 5.4	13.9 ± 4.96	$F_{(2, 143)} = 0.28$; $p = 0.755$
**Alcohol**	2.97 ± 3.94	3.83 ± 4.5	3 ± 4.01	2.48 ± 3.51	$F_{(2, 143)} = 1.06$; $p = 0.348$
**Protein**	52.2 ± 13.7	53.8 ± 14.6	48 ± 14.5	54.7 ± 11.8	$F_{(2, 143)} = 4.20$; $p = 0.017$
**Plant Protein**	50.7 ± 14.1	47.1 ± 16.4	48.1 ± 14	54.7 ± 11.8	$F_{(2, 143)} = 5.71$; $p = 0.004$
**Animal Protein**	5.6 ± 8.57	11.3 ± 9.66	8.64 ± 9.08	/	$F_{(2, 143)} = 53.7$; $p < 0.001$
**Processed Foods**	23.9 ± 14.1	25.3 ± 13.1	26.9 ± 16.7	20.8 ± 11.7	$F_{(2, 143)} = 2.12$; $p = 0.123$
**Free/Added Sugar**	14.7 ± 10.1	16 ± 10.9	16.2 ± 11.9	12.9 ± 7.53	$F_{(2, 143)} = 0.87$; $p = 0.421$

Note. Results are presented in mean (SD). F statistic calculated by Kruskal–Wallis test.
